# (*Z*)-4-[(3-Aminona­phthalen-2-yl­amino)(phen­yl)methyl­idene]-3-methyl-1-phenyl-1*H*-pyrazol-5(4*H*)-one

**DOI:** 10.1107/S1600536812034770

**Published:** 2012-08-25

**Authors:** Zhao Zhang, Xingqiang Lü, Shunsheng Zhao, Xiangrong Liu

**Affiliations:** a College of Chemical Engineering, Northwest University, Xi’an 710069, Shannxi, People’s Republic of China; b College of Chemistry and Chemical Engineering, Xian University of Science and Technology, Xi’an 710054, Shannxi, People’s Republic of China

## Abstract

The mol­ecule of the title compound, C_27_H_22_N_4_O, assumes a non-planar conformation in which the pyrazolone ring forms dihedral angles of 12.73 (11), 65.17 (6) and 49.82 (6)°, respectively, with the two benzene rings and the naphthalene ring system. In the crystal, pairs of mol­ecules are linked by inter­molecular N—H⋯N hydrogen bonds, forming dimers. The secondary amino group is involved in an intra­molecular N—H⋯O hydrogen bond.

## Related literature

For a related structure, see: Lu *et al.* (2011 ▶). For bond-length data, see: Allen *et al.* (1987 ▶). For the synthesis, see: Hennig & Mann (1988 ▶).
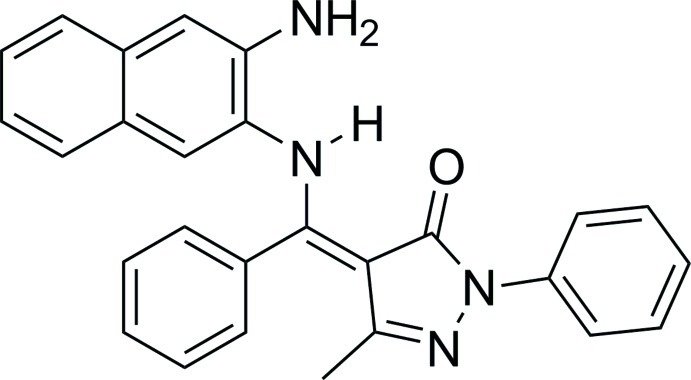


## Experimental

### 

#### Crystal data

C_27_H_22_N_4_O
*M* *_r_* = 418.49
Monoclinic, 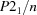

*a* = 9.8052 (14) Å
*b* = 18.041 (3) Å
*c* = 13.2193 (18) Å
β = 110.797 (2)°
*V* = 2186.0 (5) Å^3^
*Z* = 4
Mo *K*α radiation
μ = 0.08 mm^−1^
*T* = 296 K
0.31 × 0.25 × 0.24 mm

#### Data collection

Bruker SMART 1K CCD area-detector diffractometer
Absorption correction: multi-scan (*SADABS*; Sheldrick, 2004 ▶) *T*_min_ = 0.976, *T*_max_ = 0.981
10878 measured reflections
3886 independent reflections
2629 reflections with *I* > 2σ(*I*)
*R*_int_ = 0.027

#### Refinement

*R*[*F* ^2^ > 2σ(*F* ^2^)] = 0.042
*wR*(*F* ^2^) = 0.123
*S* = 1.07
3886 reflections
290 parameters
H atoms treated by a mixture of independent and constrained refinement
Δρ_max_ = 0.19 e Å^−3^
Δρ_min_ = −0.13 e Å^−3^

### 

Data collection: *SMART* (Bruker, 2001 ▶); cell refinement: *SAINT* (Bruker, 2001 ▶); data reduction: *SAINT*; program(s) used to solve structure: *SHELXS97* (Sheldrick, 2008 ▶); program(s) used to refine structure: *SHELXL97* (Sheldrick, 2008 ▶); molecular graphics: *SHELXTL* (Sheldrick, 2008 ▶); software used to prepare material for publication: *SHELXTL*.

## Supplementary Material

Crystal structure: contains datablock(s) I, global. DOI: 10.1107/S1600536812034770/ff2079sup1.cif

Structure factors: contains datablock(s) I. DOI: 10.1107/S1600536812034770/ff2079Isup2.hkl

Supplementary material file. DOI: 10.1107/S1600536812034770/ff2079Isup3.cml

Additional supplementary materials:  crystallographic information; 3D view; checkCIF report

## Figures and Tables

**Table 1:** Hydrogen-bond geometry (Å, °)

*D*—H⋯*A*	*D*—H	H⋯*A*	*D*⋯*A*	*D*—H⋯*A*
N3—H3*A*⋯O1	0.86	2.06	2.7196 (19)	133
N4—H4*A*⋯N2^i^	0.92 (2)	2.21 (2)	3.121 (2)	169.8 (18)

Symmetry code: (i) 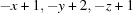.

**Fig. 1.**
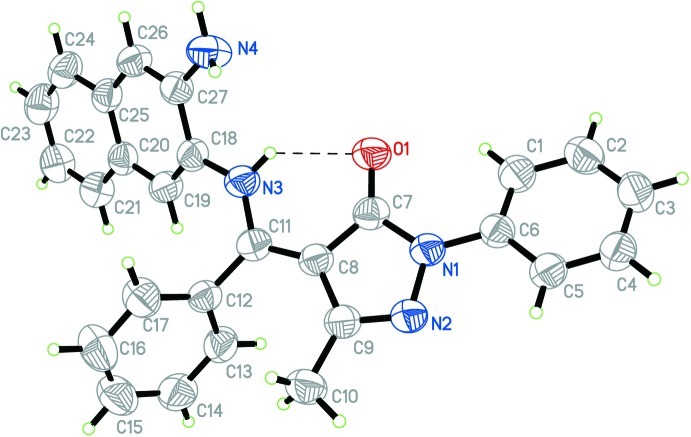
The molecular structure of (I), with atom labels and 50% probability displacement ellipsoids for non-H atoms.

**Fig. 2.**
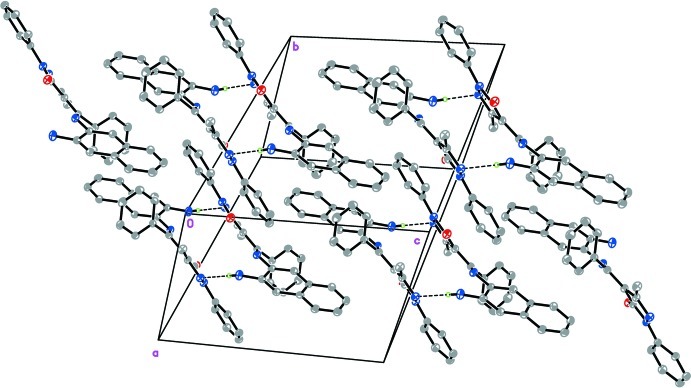
The packing of (I), showing molecules connected by N—H···N hydrogen bonds (dashed lines). H atoms not involved in hydrogen bonding have been omitted.

## supplementary crystallographic information

### Comment

Asymmetric Schiff bases attract the interest of researchers because they can form complexes with most of transition metal ions. These Schiff base complexes show excellent catalytic activity and selectivity in various reactions. Here we report the crystal structure of a novel asymmetrical Schiff base ligand (I) (Fig. 1). Bond lengths are in the range of normal values (Allen *et al.*, 1987) and are comparable to those observed in similar compounds (Lu *et al.*, 2011). The molecules of the title compound are linked by N—H···N hydrogen to form molecular pairs (Fig. 2). An intramolecular N3—H3a···O1 hydrogen bond forms an S6 ring motif.

### Experimental

The title compound was obtained according to the synthetic procedure of Hennig & Mann (1988) with some modification. 2,3-diaminonaphthalene and 4-benzoyl-3-methyl-1-phenyl-1*H*-pyrazol-5(4*H*)-one were refluxed for 2 h in a molar ratio of 1:1 in absolute ethanol to give the product. The single-crystal of suitable for X-ray diffraction was obtained by slow evaporation of its ethanolic solution of the title compound.

### Refinement

H atoms bonded to N4 were located in a difference map and refined freely. Other H atoms were positioned geometrically and refined using a riding model with C—H = 0.95–0.99 Å and N—H = 0.87 (2) Å, and with *U*_iso_(H) = 1.2 (1.5 for methyl groups) times *U*_eq_(C/N).

### Crystal data

**Table:**

C_27_H_22_N_4_O	*F*(000) = 880
*M**_r_* = 418.49	*D*_x_ = 1.272 Mg m^−^^3^
Monoclinic, *P*2_1_/*n*	Mo *K*α radiation, λ = 0.71073 Å
Hall symbol: -P 2yn	Cell parameters from 4488 reflections
*a* = 9.8052 (14) Å	θ = 1.9–25.1°
*b* = 18.041 (3) Å	µ = 0.08 mm^−^^1^
*c* = 13.2193 (18) Å	*T* = 296 K
β = 110.797 (2)°	Block, red
*V* = 2186.0 (5) Å^3^	0.31 × 0.25 × 0.24 mm
*Z* = 4	

### Data collection

**Table:**

Bruker SMART 1K CCD area-detector diffractometer	3886 independent reflections
Radiation source: fine-focus sealed tube	2629 reflections with *I* > 2σ(*I*)
Graphite monochromator	*R*_int_ = 0.027
thin–slice ω scans	θ_max_ = 25.1°, θ_min_ = 2.0°
Absorption correction: multi-scan (*SADABS*; Sheldrick, 2004)	*h* = −11→11
*T*_min_ = 0.976, *T*_max_ = 0.981	*k* = −21→15
10878 measured reflections	*l* = −15→14

### Refinement

**Table:**

Refinement on *F*^2^	Secondary atom site location: difference Fourier map
Least-squares matrix: full	Hydrogen site location: inferred from neighbouring sites
*R*[*F*^2^ > 2σ(*F*^2^)] = 0.042	H atoms treated by a mixture of independent and constrained refinement
*wR*(*F*^2^) = 0.123	*w* = 1/[σ^2^(*F*_o_^2^) + (0.0572*P*)^2^ + 0.1136*P*] where *P* = (*F*_o_^2^ + 2*F*_c_^2^)/3
*S* = 1.07	(Δ/σ)_max_ = 0.001
3886 reflections	Δρ_max_ = 0.19 e Å^−^^3^
290 parameters	Δρ_min_ = −0.13 e Å^−^^3^
0 restraints	Extinction correction: *SHELXL97* (Sheldrick, 2008), Fc^*^=kFc[1+0.001xFc^2^λ^3^/sin(2θ)]^-1/4^
Primary atom site location: structure-invariant direct methods	Extinction coefficient: 0.0046 (10)

### Special details

**Table:**

Geometry. All e.s.d.'s (except the e.s.d. in the dihedral angle between two l.s. planes) are estimated using the full covariance matrix. The cell e.s.d.'s are taken into account individually in the estimation of e.s.d.'s in distances, angles and torsion angles; correlations between e.s.d.'s in cell parameters are only used when they are defined by crystal symmetry. An approximate (isotropic) treatment of cell e.s.d.'s is used for estimating e.s.d.'s involving l.s. planes.
Refinement. Refinement of *F*^2^ against ALL reflections. The weighted *R*-factor *wR* and goodness of fit *S* are based on *F*^2^, conventional *R*-factors *R* are based on *F*, with *F* set to zero for negative *F*^2^. The threshold expression of *F*^2^ > σ(*F*^2^) is used only for calculating *R*-factors(gt) *etc*. and is not relevant to the choice of reflections for refinement. *R*-factors based on *F*^2^ are statistically about twice as large as those based on *F*, and *R*- factors based on ALL data will be even larger.

### Fractional atomic coordinates and isotropic or equivalent isotropic displacement parameters (Å^2^)

**Table:**

	*x*	*y*	*z*	*U*_iso_*/*U*_eq_	
O1	0.27552 (14)	1.00646 (7)	0.55789 (10)	0.0609 (4)	
N2	0.43043 (16)	0.85291 (8)	0.47774 (12)	0.0549 (4)	
N1	0.31277 (16)	0.89991 (8)	0.46993 (11)	0.0516 (4)	
N3	0.51820 (16)	1.03547 (8)	0.73333 (11)	0.0540 (4)	
H3A	0.4486	1.0534	0.6789	0.065*	
C18	0.55379 (19)	1.07787 (9)	0.82950 (14)	0.0490 (4)	
C26	0.5429 (2)	1.19826 (10)	0.90501 (15)	0.0556 (5)	
H26A	0.5190	1.2483	0.8961	0.067*	
C27	0.52031 (19)	1.15528 (10)	0.81475 (14)	0.0500 (4)	
C25	0.60116 (19)	1.16913 (10)	1.01105 (15)	0.0523 (5)	
C12	0.71102 (19)	0.94103 (9)	0.79235 (13)	0.0476 (4)	
C11	0.57512 (19)	0.97179 (9)	0.71287 (14)	0.0482 (4)	
C8	0.50078 (19)	0.93546 (9)	0.61583 (13)	0.0481 (4)	
C5	0.1637 (2)	0.83859 (9)	0.30338 (14)	0.0541 (5)	
H5A	0.2457	0.8137	0.3014	0.065*	
C6	0.1758 (2)	0.88711 (9)	0.38791 (14)	0.0488 (4)	
C7	0.3538 (2)	0.95422 (10)	0.54880 (13)	0.0495 (4)	
N4	0.4582 (2)	1.18265 (11)	0.71068 (14)	0.0656 (5)	
C19	0.60730 (19)	1.04834 (10)	0.93090 (14)	0.0532 (5)	
H19A	0.6266	0.9978	0.9389	0.064*	
C20	0.63419 (19)	1.09256 (10)	1.02421 (14)	0.0504 (5)	
C21	0.6924 (2)	1.06300 (11)	1.12955 (15)	0.0616 (5)	
H21A	0.7117	1.0125	1.1385	0.074*	
C3	−0.0920 (2)	0.86374 (11)	0.22376 (17)	0.0646 (5)	
H3B	−0.1812	0.8567	0.1681	0.078*	
C9	0.5400 (2)	0.87347 (9)	0.56375 (14)	0.0507 (5)	
C13	0.7088 (2)	0.87261 (10)	0.83947 (15)	0.0612 (5)	
H13A	0.6213	0.8469	0.8229	0.073*	
C1	0.0515 (2)	0.92247 (10)	0.38980 (15)	0.0606 (5)	
H1A	0.0572	0.9545	0.4463	0.073*	
C23	0.6896 (2)	1.18263 (13)	1.20526 (17)	0.0721 (6)	
H23A	0.7097	1.2127	1.2659	0.087*	
C4	0.0309 (2)	0.82743 (10)	0.22284 (15)	0.0610 (5)	
H4C	0.0239	0.7948	0.1668	0.073*	
C24	0.6299 (2)	1.21312 (11)	1.10469 (16)	0.0646 (5)	
H24A	0.6079	1.2634	1.0977	0.077*	
C17	0.8418 (2)	0.97844 (11)	0.81943 (16)	0.0638 (5)	
H17A	0.8446	1.0250	0.7900	0.077*	
C10	0.6841 (2)	0.83662 (11)	0.58793 (16)	0.0651 (6)	
H10A	0.6744	0.7966	0.5379	0.098*	
H10B	0.7172	0.8175	0.6604	0.098*	
H10C	0.7535	0.8720	0.5810	0.098*	
C22	0.7211 (2)	1.10693 (12)	1.21862 (16)	0.0671 (6)	
H22A	0.7613	1.0867	1.2876	0.081*	
C2	−0.0809 (2)	0.91046 (11)	0.30816 (17)	0.0660 (6)	
H2B	−0.1638	0.9344	0.3104	0.079*	
C14	0.8356 (3)	0.84252 (11)	0.91075 (16)	0.0726 (6)	
H14A	0.8332	0.7969	0.9430	0.087*	
C16	0.9680 (2)	0.94749 (14)	0.88954 (18)	0.0805 (6)	
H16A	1.0559	0.9729	0.9067	0.097*	
C15	0.9647 (3)	0.87940 (14)	0.93425 (18)	0.0787 (6)	
H15A	1.0506	0.8582	0.9807	0.094*	
H4A	0.497 (2)	1.1671 (11)	0.6603 (17)	0.083 (7)*	
H4B	0.436 (3)	1.2320 (14)	0.7045 (17)	0.098 (8)*	

### Atomic displacement parameters (Å^2^)

**Table:**

	*U*^11^	*U*^22^	*U*^33^	*U*^12^	*U*^13^	*U*^23^
O1	0.0635 (9)	0.0555 (8)	0.0600 (8)	0.0159 (6)	0.0173 (7)	−0.0065 (6)
N2	0.0542 (10)	0.0522 (9)	0.0562 (10)	0.0090 (7)	0.0168 (8)	−0.0072 (7)
N1	0.0522 (10)	0.0506 (9)	0.0494 (9)	0.0094 (7)	0.0147 (8)	−0.0073 (7)
N3	0.0600 (10)	0.0483 (9)	0.0482 (9)	0.0088 (7)	0.0124 (8)	−0.0048 (7)
C18	0.0474 (11)	0.0483 (10)	0.0510 (11)	−0.0011 (8)	0.0171 (9)	−0.0080 (8)
C26	0.0581 (12)	0.0463 (10)	0.0633 (12)	0.0025 (8)	0.0227 (10)	−0.0054 (9)
C27	0.0468 (11)	0.0509 (11)	0.0529 (11)	0.0013 (8)	0.0183 (9)	−0.0017 (9)
C25	0.0474 (11)	0.0527 (11)	0.0585 (12)	−0.0064 (8)	0.0211 (9)	−0.0085 (9)
C12	0.0521 (11)	0.0445 (10)	0.0469 (10)	0.0020 (8)	0.0183 (9)	−0.0022 (8)
C11	0.0522 (11)	0.0445 (10)	0.0504 (11)	0.0006 (8)	0.0213 (9)	0.0009 (8)
C8	0.0530 (11)	0.0444 (10)	0.0453 (10)	0.0053 (8)	0.0156 (9)	−0.0016 (8)
C5	0.0588 (13)	0.0497 (11)	0.0527 (11)	0.0026 (9)	0.0183 (10)	−0.0022 (9)
C6	0.0525 (11)	0.0461 (10)	0.0467 (10)	0.0015 (8)	0.0160 (9)	0.0030 (8)
C7	0.0591 (12)	0.0476 (10)	0.0438 (10)	0.0048 (9)	0.0208 (9)	−0.0003 (8)
N4	0.0803 (13)	0.0607 (12)	0.0561 (11)	0.0229 (9)	0.0246 (10)	0.0045 (9)
C19	0.0566 (12)	0.0461 (10)	0.0570 (12)	0.0013 (8)	0.0205 (10)	−0.0016 (9)
C20	0.0452 (11)	0.0538 (11)	0.0527 (11)	−0.0046 (8)	0.0180 (9)	−0.0050 (9)
C21	0.0644 (13)	0.0646 (13)	0.0579 (12)	−0.0017 (10)	0.0245 (10)	−0.0017 (10)
C3	0.0579 (13)	0.0642 (13)	0.0609 (13)	−0.0016 (10)	0.0078 (10)	0.0031 (10)
C9	0.0551 (12)	0.0476 (10)	0.0488 (11)	0.0056 (8)	0.0175 (10)	0.0000 (8)
C13	0.0642 (13)	0.0487 (11)	0.0619 (13)	−0.0055 (9)	0.0115 (10)	0.0026 (10)
C1	0.0603 (13)	0.0642 (12)	0.0562 (12)	0.0064 (10)	0.0192 (11)	−0.0059 (10)
C23	0.0765 (16)	0.0817 (16)	0.0611 (14)	−0.0114 (12)	0.0280 (12)	−0.0202 (12)
C4	0.0683 (14)	0.0551 (12)	0.0541 (12)	−0.0011 (10)	0.0150 (11)	−0.0044 (9)
C24	0.0716 (14)	0.0604 (12)	0.0640 (14)	−0.0069 (10)	0.0269 (11)	−0.0162 (10)
C17	0.0571 (13)	0.0641 (12)	0.0697 (14)	−0.0029 (10)	0.0219 (11)	0.0122 (10)
C10	0.0588 (13)	0.0683 (13)	0.0659 (13)	0.0173 (10)	0.0191 (11)	−0.0072 (10)
C22	0.0686 (14)	0.0826 (16)	0.0513 (12)	−0.0031 (11)	0.0226 (11)	−0.0023 (11)
C2	0.0564 (13)	0.0692 (13)	0.0690 (14)	0.0094 (10)	0.0183 (11)	0.0023 (11)
C14	0.0867 (18)	0.0538 (12)	0.0636 (14)	0.0054 (11)	0.0098 (12)	0.0096 (10)
C16	0.0520 (14)	0.0979 (18)	0.0852 (16)	−0.0040 (12)	0.0167 (12)	0.0146 (14)
C15	0.0643 (15)	0.0876 (17)	0.0703 (15)	0.0164 (12)	0.0065 (12)	0.0072 (13)

### Geometric parameters (Å, º)

**Table:**

O1—C7	1.2480 (19)	C19—H19A	0.9300
N2—C9	1.310 (2)	C20—C21	1.409 (2)
N2—N1	1.4056 (19)	C21—C22	1.363 (3)
N1—C7	1.382 (2)	C21—H21A	0.9300
N1—C6	1.414 (2)	C3—C2	1.371 (3)
N3—C11	1.346 (2)	C3—C4	1.376 (3)
N3—C18	1.417 (2)	C3—H3B	0.9300
N3—H3A	0.8600	C9—C10	1.490 (2)
C18—C19	1.362 (2)	C13—C14	1.377 (3)
C18—C27	1.432 (2)	C13—H13A	0.9300
C26—C27	1.373 (2)	C1—C2	1.379 (3)
C26—C25	1.414 (2)	C1—H1A	0.9300
C26—H26A	0.9300	C23—C24	1.364 (3)
C27—N4	1.383 (2)	C23—C22	1.398 (3)
C25—C24	1.412 (2)	C23—H23A	0.9300
C25—C20	1.415 (2)	C4—H4C	0.9300
C12—C17	1.379 (3)	C24—H24A	0.9300
C12—C13	1.386 (2)	C17—C16	1.374 (3)
C12—C11	1.481 (2)	C17—H17A	0.9300
C11—C8	1.394 (2)	C10—H10A	0.9600
C8—C9	1.436 (2)	C10—H10B	0.9600
C8—C7	1.439 (2)	C10—H10C	0.9600
C5—C4	1.373 (3)	C22—H22A	0.9300
C5—C6	1.391 (2)	C2—H2B	0.9300
C5—H5A	0.9300	C14—C15	1.365 (3)
C6—C1	1.384 (2)	C14—H14A	0.9300
N4—H4A	0.92 (2)	C16—C15	1.368 (3)
N4—H4B	0.91 (2)	C16—H16A	0.9300
C19—C20	1.413 (2)	C15—H15A	0.9300
			
C9—N2—N1	106.96 (14)	C22—C21—H21A	119.3
C7—N1—N2	111.14 (15)	C20—C21—H21A	119.3
C7—N1—C6	129.56 (14)	C2—C3—C4	118.88 (19)
N2—N1—C6	119.30 (14)	C2—C3—H3B	120.6
C11—N3—C18	130.80 (15)	C4—C3—H3B	120.6
C11—N3—H3A	114.6	N2—C9—C8	111.09 (16)
C18—N3—H3A	114.6	N2—C9—C10	118.87 (16)
C19—C18—N3	123.89 (15)	C8—C9—C10	129.78 (17)
C19—C18—C27	120.33 (16)	C14—C13—C12	120.25 (19)
N3—C18—C27	115.64 (15)	C14—C13—H13A	119.9
C27—C26—C25	122.37 (16)	C12—C13—H13A	119.9
C27—C26—H26A	118.8	C2—C1—C6	120.25 (18)
C25—C26—H26A	118.8	C2—C1—H1A	119.9
C26—C27—N4	122.76 (17)	C6—C1—H1A	119.9
C26—C27—C18	118.27 (16)	C24—C23—C22	121.09 (19)
N4—C27—C18	118.84 (16)	C24—C23—H23A	119.5
C24—C25—C26	123.02 (17)	C22—C23—H23A	119.5
C24—C25—C20	118.36 (17)	C5—C4—C3	121.06 (18)
C26—C25—C20	118.61 (16)	C5—C4—H4C	119.5
C17—C12—C13	118.74 (18)	C3—C4—H4C	119.5
C17—C12—C11	121.35 (16)	C23—C24—C25	120.72 (19)
C13—C12—C11	119.90 (16)	C23—C24—H24A	119.6
N3—C11—C8	117.88 (16)	C25—C24—H24A	119.6
N3—C11—C12	120.67 (15)	C16—C17—C12	120.53 (19)
C8—C11—C12	121.44 (15)	C16—C17—H17A	119.7
C11—C8—C9	131.77 (17)	C12—C17—H17A	119.7
C11—C8—C7	122.58 (15)	C9—C10—H10A	109.5
C9—C8—C7	105.55 (15)	C9—C10—H10B	109.5
C4—C5—C6	120.14 (17)	H10A—C10—H10B	109.5
C4—C5—H5A	119.9	C9—C10—H10C	109.5
C6—C5—H5A	119.9	H10A—C10—H10C	109.5
C1—C6—C5	118.70 (18)	H10B—C10—H10C	109.5
C1—C6—N1	120.94 (16)	C21—C22—C23	119.4 (2)
C5—C6—N1	120.37 (15)	C21—C22—H22A	120.3
O1—C7—N1	125.71 (17)	C23—C22—H22A	120.3
O1—C7—C8	129.36 (16)	C3—C2—C1	120.95 (19)
N1—C7—C8	104.92 (14)	C3—C2—H2B	119.5
C27—N4—H4A	117.6 (13)	C1—C2—H2B	119.5
C27—N4—H4B	116.4 (14)	C15—C14—C13	120.2 (2)
H4A—N4—H4B	112.3 (19)	C15—C14—H14A	119.9
C18—C19—C20	121.69 (16)	C13—C14—H14A	119.9
C18—C19—H19A	119.2	C15—C16—C17	120.2 (2)
C20—C19—H19A	119.2	C15—C16—H16A	119.9
C21—C20—C19	122.25 (17)	C17—C16—H16A	119.9
C21—C20—C25	119.06 (16)	C14—C15—C16	120.1 (2)
C19—C20—C25	118.69 (16)	C14—C15—H15A	120.0
C22—C21—C20	121.36 (19)	C16—C15—H15A	120.0

### Hydrogen-bond geometry (Å, º)

**Table:**

*D*—H···*A*	*D*—H	H···*A*	*D*···*A*	*D*—H···*A*
N3—H3*A*···O1	0.86	2.06	2.7196 (19)	133
N4—H4*A*···N2^i^	0.92 (2)	2.21 (2)	3.121 (2)	169.8 (18)

Symmetry code: (i) −*x*+1, −*y*+2, −*z*+1.
